# Nanowire electrodes for high-density stimulation and measurement of neural circuits

**DOI:** 10.3389/fncir.2013.00038

**Published:** 2013-03-12

**Authors:** Jacob T. Robinson, Marsela Jorgolli, Hongkun Park

**Affiliations:** ^1^Departments of Electrical and Computer Engineering and Bioengineering, Rice UniversityHouston, TX, USA; ^2^Department of Physics, Harvard UniversityCambridge, MA, USA; ^3^Department of Chemistry and Chemical Biology, Harvard UniversityCambridge, MA, USA

**Keywords:** brain machine interface (BMI), nanotechnology, nanowires, neuroengineering, electrophysiology

## Abstract

Brain-machine interfaces (BMIs) that can precisely monitor and control neural activity will likely require new hardware with improved resolution and specificity. New nanofabricated electrodes with feature sizes and densities comparable to neural circuits may lead to such improvements. In this perspective, we review the recent development of vertical nanowire (NW) electrodes that could provide highly parallel single-cell recording and stimulation for future BMIs. We compare the advantages of these devices and discuss some of the technical challenges that must be overcome for this technology to become a platform for next-generation closed-loop BMIs.

Today, brain-machines interfaces (BMIs) enable users to manipulate prosthetic limbs and computer interfaces by monitoring and processing their neural activity (Donoghue et al., 2007; Simeral et al., 2011). BMIs can also be used to treat neurological disorders such as Parkinson's disease (Volkmann, 2004), obsessive compulsive disorder (Bourne et al., 2012), and depression (Howland et al., 2011) by applying voltage or current pulses to specific regions deep within the brain—a treatment known as deep brain stimulation (DBS). As remarkable as today's BMI technology is, it is in many ways in its infancy. Future technology will seek to improve the precision with which external devices can be manipulated and the specificity of stimulation to the level of individual cells. These improvements will help expand the capabilities of neural prosthetics and extend the range of disorders that can be treated using DBS (Donoghue et al., 2007). To achieve these goals, the next generation of BMIs will need improved resolution for measurement and stimulation, as well as the ability to adjust their spatial and temporal stimulation patterns based on the current state of the neural activity (the devices with this latter capability are often termed “closed-loop” BMIs) (Stanslaski et al., 2012).

Currently, the large size and small number of electrodes in BMIs limits their stimulation and measurement resolution. State-of-the-art devices for DBS typically have 4–8 millimeter-sized electrodes (Stanslaski et al., 2012), whereas BMIs for neural recording typically use a few dozen electrodes that are 10–100 microns in diameter (Hochberg et al., 2006; Donoghue et al., 2007; Du et al., 2011) (Figure 1A). This density and feature size is a far cry from that of the human brain, which contains approximately one hundred billion neurons, each with diameter as fine as 10 microns (Williams and Herrup, 1988). In fact, a single square millimeter of brain tissue contains approximately one million neurons (Williams and Herrup, 1988). To match this number and density, future BMIs must feature smaller and denser electrode arrays in order to precisely monitor and control neural circuit activity. Furthermore, smaller electrodes (<1 micron in diameter) may also enable the recording of intracellular electrical signals of individual neurons (Figure 1A): compared to extracellular recording, these intracellular measurements will have improved signal to noise ratio and enable a clear cell-to-electrode registry (Figures 1B–D). Importantly, the improved signal to noise ratio also enables intracellular electrodes to record subthreshold neural activity (e.g., postsynaptic potentials) that can be used to determine the strength of synaptic connectivity.

**Figure 1:**
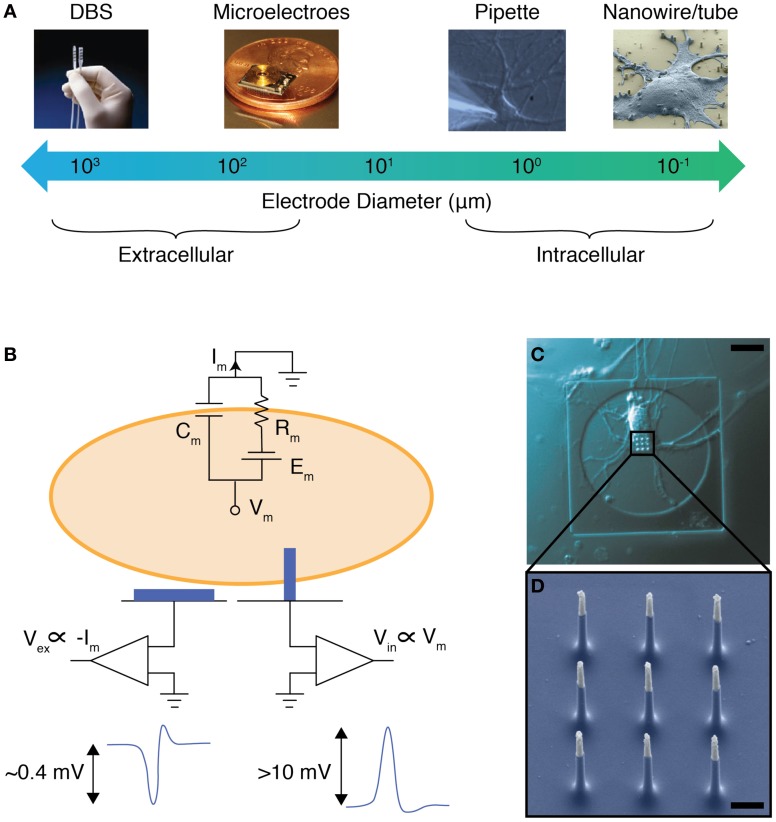
**NWs as intracellular electrodes. (A)** The electrode diameters for various methods to record and stimulate neural activity. Electrodes with diameters less than 1 micron can be used as intracellular probes. Photo credits: DBS—EPDA.com, Microelectrodes—microsystems.utah.edu. **(B)** Equivalent circuit model for a cell on top of an extracellular (left) and intracellular (right) electrode. The membrane resistance, capacitance, and Nernst potential is shown as R_*m*_, C_*m*_, and E_*m*_, respectively. The voltage recorded extracellularly (V_ex_) is proportional to I_*m*_, and typically has a magnitude of 0.4 mV for a neuronal action potential. The voltage recorded intracellularly (V_in_), however, is proportional to V_*m*_, and typically has a magnitude of greater than 10 mV for a neuronal action potential. **(C)** Optical microscope image of a rat cortical neuron grown on top of a vertical NW electrode, scale bar 10 microns. **(D)** Scanning electron micrograph of a set of vertical NWs, scale bar 1 micron. [**(C)** and **(D)** adapted from Robinson et al. (2012)].

Fortunately devices that match the feature size and density of neural circuits are routinely fabricated on silicon using contemporary nanofabrication techniques (Arden, 2002). This observation highlights the future role for semiconductor fabrication and nanotechnology as a platform for high-precision BMIs. Recently, these nanofabrication techniques have been used to create vertical nanowires (NWs) and nanotubes that can intracellularly stimulate and record the activity of neurons and other electrically active cells. Here we review this technology, highlight the characteristics that make NW electrodes an attractive platform for future BMIs, and comment on some of the challenges that face the development of these next-generation devices.

## NWs as intracellular electrodes

**Figure 2:**
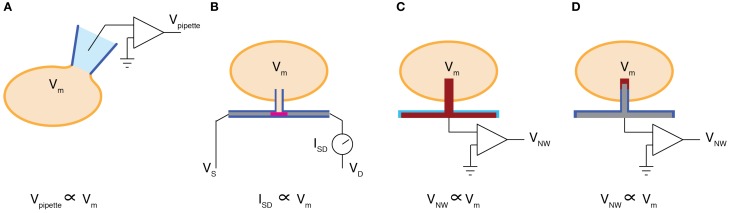
**Intracellular recording methods. (A)** Whole cell patch pipette configuration measures a voltage (V_pipette_) proportional to the membrane potential (V_*m*_). **(B)** A vertical glass nanotube (blue) is grown on top of an FET (pink) that lies within an insulated NW (gray). When the nanotube penetrates the cellular membrane, the membrane potential can be measured as a change in the source-drain current (I_SD_). **(C)** A platinum NW (red) is deposited on top of a platinum electrode (red) that is insulated by silicon nitride (blue). The voltage recorded at the NW (V_NW_) is then proportional to the membrane potential. **(D)** A silicon NW (gray) insulated by glass (blue) is capped with a metallic film such as platinum (red). Similarly to **(C)**, V_NW_ is proportional to V_*m*_, however in this configuration the NW sidewalls are insulated by glass, improving the amplitude of the measured signal and proving a surface for cell membrane fusion.

The electrical activity of neurons is most directly measured as the electrical potential across the cellular membrane. As a result, intracellular electrodes that can directly measure the membrane potential are widely considered the “gold standard” in neuronal recording. These high fidelity measurements, however, come at a cost. To monitor intracellular signals via traditional patch-clamp methods, glass micropipettes must be carefully aligned to the cellular membrane using manually controlled micromanipulators. Once the pipette is placed in contact with the cell, an intracellular electrical connection can be formed either by slowly driving the pipette through the cellular membrane or by applying negative pressure to seal the membrane against the pipette and then rupturing the circumscribed patch using a current pulse or a rapid impulse of negative pressure (Figure 2A). While this painstaking process is a commonly performed procedure, it is not scalable. As a result, today's BMIs are based on extracellular recording techniques that sacrifice the high fidelity of intracellular measurements for the sake of scalability. For instance, contemporary extracellular electrodes have array sizes approaching one hundred electrodes and can simultaneously measure the activity of dozens of individual neurons (Hochberg et al., 2006; Donoghue et al., 2007; Du et al., 2011).

While extracellular electrodes succeed in monitoring large numbers of neurons, there are potential challenges for using these devices as the basis for closed-loop BMIs. For instance, a single extracellular electrode records the spiking activity of many nearby cells. This makes it difficult to identify the activity that corresponds to each individual neuron. While a variety of computational techniques can be used to sort the recorded spikes based on their waveforms, this process typically requires a training period of several minutes, and must be repeated on a daily basis as the electrical coupling between the cells and extracellular electrode changes (Donoghue et al., 2007). Furthermore, typical signal to noise ratios are less than 10:1 (Hochberg et al., 2006; Donoghue et al., 2007; Du et al., 2011), and therefore slight degradation of the signal amplitude during chronic implantation leads to a gradual reduction in the number of individual neurons that can be recorded (Dickey et al., 2009). Stimulation using extracellular electrodes also lacks precise cell-to-electrode registry. During voltage or current pulses from an extracellular electrode, many cells in the vicinity of the electrode will be activated. This shortcoming ultimately limits the spatial accuracy of stimuli applied via extracellular probes.

To improve the cell-to-electrode registration, the size of the electrodes can be scaled down so that an individual electrode can interface to at most a single neuron (Figure 1A). This is the approach taken recently for vertical NW electrodes. Three recent papers have shown that these electrodes can be made small enough to penetrate the cellular membrane without compromising cell viability, and record or stimulate individual cells (Duan et al., 2012; Robinson et al., 2012; Xie et al., 2012). Thus silicon-based intracellular electrodes can provide both precise cell-to-electrode registration as well as large signal-to-noise ratios typically reserved for patch clamp recordings. Importantly, these NW devices can be fabricated using semiconductor nanofabrication techniques that can be scaled up to produce tens of thousands of recording sites in a single fabrication run, making this technology a potential platform for next generation BMIs requiring an increased number of electrodes.

Although the three recent demonstrations of vertical NW electrodes employed different fabrication strategies, each reported successful intracellular electrical measurements. Duan et al. used electron beam lithography to define nanoscale gold islands on the gate region of NW field-effect transistors (FETs). They used these islands as precursors for germanium (Ge) NW vapor-liquid-solid (VLS) growth (Duan et al., 2012). The Ge NWs were then coated with SiO_2 using atomic layer deposition and the Ge core was subsequently etched away using Hydrogen Peroxide. This process left a nanoscale glass tube leading the gate region of the NW FET (Figure 2B). The authors showed that when this glass nanotube penetrated the cellular membrane of a cardiomyocyte, the intracellular membrane potential could be recorded. In this configuration the cardiomyocyte membrane potential gates the NW FET such that the source-drain conductance maps to the intracellular membrane potential. One advantage of using the FET conductance to transduce the membrane potential is that the gain of the NW FET can be used to amplify the measured signal. An alternative strategy pursued by Robinson et al. used plasma etching to micromachine solid silicon (Si) NWs out of highly conductive silicon-on-insulator wafers (Robinson et al., 2012). The resulting Si NWs were then insulated by a thermally grown silicon oxide that was subsequently removed from the Si NW tips. The Si NW tips were coated with an evaporated platinum or gold film (Figure 2D). Unlike NW FETs, this approach requires amplifying electronics to boost the signal recorded by the Si NWs. At the same time, however, the Si NW electrodes can also be used to stimulate electrical activity by injecting current into the cell, thereby evoking neuronal action potentials on demand. Using the stimulation capabilities of the solid Si NWs, functional circuits can be reconstructed by systematically stimulating individual electrodes and recording the resulting response at another cell (Robinson et al., 2012). A third fabrication technique was employed by Xie et al. who used focused ion beams to deposit 100 nm diameter platinum NWs on top of planar platinum electrodes (Xie et al., 2012) (Figure 2C). The functionality of these devices were similar to those reported by Robinson et al. although Xie et al. used their device to monitor mitotic cardiac cells as opposed to primary neurons.

For each NW electrode device, care must be taken to secure a stable intracellular measurement. To improve the stability of the cell-electrode interface and promote NW penetration, Duan et al. coated their devices with a phospholipid (Duan et al., 2012). One drawback to this method is that the phospholipid coating prevents cells from being cultured directly on top of the electrodes. As a result, to test these devices cells were grown on a separate PDMS substrate, inverted, and aligned atop the devices for measurement. Alternatively, Robinson et al. and Xie et al. did not use a surface treatment to promote penetration and reported intracellular recordings in cells grown directly on top of the devices. Both groups reported that, at times, voltage pulses are required to permeabilize the membrane covering the electrode in order to achieve intracellular electrical coupling. Xie et al. showed that over time, the permeabilized membrane recovers; however, repeated application of voltage pulses can restore the intracellular electrical coupling. The observed time scale of this recovery is consistent with the kinetics of cell membrane electroporation for biomolecule delivery (Saulis et al., 1991).

## Future challenges for NW electrodes *in vivo*

While electrically facilitated membrane permeabilization is adequate for *in vitro* studies, a long-term *in vivo* interface will likely require alternative approaches to stabilize the NW-cell interface. Potential surface treatments include the phospholipid coating used by Duan et al. and biomimetic surfaces developed by Almquist and Melosh (2010). While these methods have been successful in securing stable interfaces between cells and nanostructures, future studies should investigate their long term stability and biocompatibility *in vivo*.

In addition to stabilizing the cellular interface, future *in vivo* devices must also deal with the motion of the brain resulting from the expanding and contracting vasculature (Enzmann and Pelc, 1992). Improving the flexibility of the NW electrodes may help them function in living tissue like the brain. Recently Tian et al. have taken steps in this direction by embedding NW FETs in a flexible polymer matrix and releasing them from the rigid substrate (Tian et al., 2012). While these devices, which have only recently been reported, have yet to be used for intracellular measurements, this approach may allow intracellular electrodes to remain within the cell while the tissue moves. Supporting this idea are reports that vertical NWs can pin neurons in place and inhibit them from migrating away from NW electrodes (Xie et al., 2010). Such effects may help secure intracellular coupling *in vivo*. An alternative approach may be to imbed vertical silicon NWs in a PDMS or other polymeric matrix that can be peeled off the silicon substrate. Such methods have recently been used to create flexible photonic crystal cavities based on semiconductor NWs, and may be adapted to support vertical NW electrodes (Yu et al., 2013).

Another challenge to using NW electrodes *in vivo* is recording beyond the layer of dead cells and protective glia that surround implanted electrodes. Studies have reported the thickness of the dead cell layers to be approximately 40 microns (Chia and Levene, 2009). Therefore, to access healthy cells, the NWs electrodes must penetrate through this dead layer. One approach is to make long electrode shanks tipped with NW electrodes. Alternatively very high aspect ratio NWs can be fabricated using deep reactive ion etching and oxide thinning processes (Morton et al., 2008). Such high aspect ratio NWs will have increased flexibility (Li et al., 2009), which may help accommodate tissue movement.

Another concern for closed loop BMIs is the potential for crosstalk between stimulation and recording electrodes that could interfere with the feedback algorithms. Potential solutions to this problem fall primarily into two categories: (1) Recording hardware and/or software can be modified to reduce the crosstalk using techniques such as blanking, spectral filtering, or common mode rejection (Stanslaski et al., 2012) or (2) Stimulation can be performed optically using channel-rhodopsin (Erickson et al., 2008) or near-IR stimulation (Wells et al., 2005). The advantage of modifying the recording electronics is that there is no need to genetically modify neuronal populations or to employ optical sources and components. However, the added electronic elements needed to reduce crosstalk can increase the size and power consumption of the BMI. Optical stimulation, on the other hand, typically produces negligible electronic crosstalk, simplifying the design requirements and lowering the power consumption of the recording electronics.

Finally, to achieve the thousands to millions of recording sites that will be desired for future BMIs the number of electrodes must be increased. The NW electrodes described here have relied on a direct electrical connection to each electrode. This method, requiring a dedicated wire for each recording/stimulation site, is impractical for devices with large numbers of electrodes. Fortunately solutions to this “interconnect problem” have already been solved for semiconductor electronics. Image sensors, for instance, contain millions of pixels that can be read using only a few dozen connections. This reduced number of contacts is achieved by interleaving data from several pixels and transmitting it over a single wire. This multiplexing process can be implemented on-chip using complementary metal oxide semiconductor (CMOS) technology (Lei et al., 2011). Yet another advantage of silicon-based electrodes is that they can be readily coupled to back-side CMOS electronics using bonding or other post-processing methods such as those used for extracellular electrodes (Kim et al., 2009). The combination of NW electrodes and CMOS multiplexing will allow high-resolution BMIs to be created on a compact monolithic platform.

## Conclusions

In the future, closed-loop BMIs will see improvements both to the hardware that interfaces to the neural circuits, and to the software that drives their activity. Ultimately this hardware may be able to stimulate and record the activity of thousands to millions of neurons with single-cell resolution. The high resolution combined with algorithms to identify the state of the neural circuit and predict its response to stimuli would provide a basis for new classes of BMIs that can accurately control neural circuits and translate this recorded activity into accurate manipulation of prosthetic devices. While there is currently no technology that can achieve such a high-resolution electrical interface, NW-based electrodes have made considerable progress toward this goal *in vitro*. To take the next step and employ nanotechnology for BMIs, efforts must be taken to improve the stability of the interface, flexibility of the electrodes, and compatibility with layers of dead cells and glia that accompany surgical implantation of electrodes. With these improvements, NW electrodes may become the preferred technology for high-resolution BMIs.

### Conflict of interest statement

The authors declare that the research was conducted in the absence of any commercial or financial relationships that could be construed as a potential conflict of interest.
